# Occurrence of some rare earth elements in vineyard soils under semiarid Mediterranean environment

**DOI:** 10.1007/s10661-022-09956-z

**Published:** 2022-04-07

**Authors:** Jiménez-Ballesta R., Bravo S., Amorós J.A., Pérez-de-los-Reyes C., García-Pradas J., Sanchez M., García-Navarro F.J.

**Affiliations:** 1 Deparment of Geology and Geochemistry, Autónoma University of Madrid, Madrid, Spain; 2 Higher Technical School Agricultural Engineers of Ciudad Real, University of Castilla-La Mancha, Ciudad Real, Spain

**Keywords:** Lanthanides, Spatial variability, *Vitis Vinifera L*., La Mancha, Calcareous soils, Contamination assessment

## Abstract

A comprehensive investigation has been carried out into the concentrations of a range of REEs (neodymium Nd, cerium Ce, lanthanum La, yttrium Y, scandium Sc) in soils of vineyards belonging to the protected denomination of origin (PDO) Valdepeñas (Central Spain). The mean concentrations (expressed in mg kg^−1^) are Ce 70.6, Nd 32.9, La 36.2, Y 21.6, and Sc 13.7 in surface horizons (Ap), while in subsurface horizons (Bt or Bw and some Ck), the values are Ce 67.6, Nd 31.8, La 34.4, Y 19.6, and Sc 13.9. The relative abundance in these soils is Ce > La > Nd > Y > Sc in both the surface and subsurface horizons. These values are close to, or slightly higher than, the regional levels but similar to national and global averages, although relatively high values have been detected at certain sampling points. Another aim was to explain the spatial variations in these elements within the territory under study. It was found that the spatial variations are due to the nature of the parent materials and the pedogenetic processes, although the sparse spatial distribution patterns with prominent anomalies are interpreted arising from anthropogenic sources (fertilization). However, these anomalies did not present any environmental risk in the studied zone.

## Introduction

The Rare Earth Elements (REEs) are a group of elements (not as rare in geological abundance) that are widely distributed in the environment (Chen, 2011; Migaszewski et al., 2019; Vural, 2020; Kang & Kang, 2020). According to the International Union of Pure and Applied Chemistry (IUPAC, 2005), they include 17 trivalent elements with very similar chemical and physical properties. REEs include the 15 lanthanides: La to Lu; moreover, Sc and Y are included as well. REEs are generally subdivided into three subgroups: the light (LREE, from La to Eu), middle (from Sa to Ho), and heavy (HREE, from Gd to Lu) REEs.

REEs have been used in the identification of weathering processes, edaphoclimatic changes, and soil evolution (Aide & Smith, 2001; Aide & Pavich, 2002; Aide & Smith-Aide, 2003; Aide, 2005; Sterckeman et al., 2006, Laveuf et al., 2008; Laveuf & Cornu, 2009; Ramos et al., 2016). Mihajlovic and Rinklebe (2018) reviewed the knowledge about REE contents and potential mobilization in different soil profiles in Germany and indicated that the contents in REEs tend to decrease according to the parent materials as follows: carbonatite > basalt > orthogneiss > clay slate > loess > sandstone > Pleistocene and Holocene sediments > organic material. In the case of limestone rocks, Abdel-Haleel et al. (2001) indicated that the contents of REEs (in mg kg^−1^) are Ce 10.4, La 19.5, and Sc 0.2.

Although the contents of REEs in soils are low (Ramos et al., 2016), they provide valuable agronomic information (Censi et al., 2014; Drivelos et al., 2014; Pepi et al., 2016a, b). The contents of REEs in plants are even lower than those in soils, even though some reports have highlighted the stimulation of plant growth in the presence of REE fertilizers (Ji et al., 1985; Pang et al., 2002). These elements can accumulate in soils following anthropogenic inputs because of the low mobility of these elements and its consequent persistence (Cao et al., 2000; Liang et al., 2005). For this reason, the addition of fertilizers to soils may lead to an accumulation of REEs in the soils themselves (Otero et al., 2005).

The study of the environmental geochemistry of REEs in viticulture in a general sense, and in vineyard soils in particular, has attracted increasing attention in the last two decades (Mihucz et al., 2006; Rossano et al., 2007; Bertoldi et al., 2009, 2011; Censi et al., 2014; Pisciotta et al., 2017; Pepi et al., 2016a, b; Punturo et al., 2018; Pepi et al., 2020). These studies have shown that vineyard soils can contain high concentrations of both heavy metals and REEs and this finding has encouraged studies aimed at identifying the contents of such elements and their influence on wine quality.

The mineral content of a given wine is generally understood to start from the vineyard soil via grapevine roots. For this reason, some authors, such as Greenough et al. (1997) and Martins et al. (2014), have used REEs (along with other elements) for the identification of potential fingerprints in order to determine the authenticity of wine. However, during the vegetative development of the vine and the corresponding winemaking process, the contents of these elements may increase for anthropogenic reasons, e.g., soil amendments, fertilizers, pesticides, irrigation water, or due to winemaking and enological processing aids, additives, and even atmospheric pollution (Pang et al., 2002; Gomez et al., 2004; Volpe et al., 2009). It is known that the presence of REEs in bentonites can lead to the presence of these elements in wine (Catarino et al., 2008a, b). The results of other studies revealed that certain viticultural practices can alter the contents of metals and REEs, which makes it more difficult to determine the possible relationship between soils and wines (Almeida & Vasconcelos, 2003; Catarino et al., 2008a, b).

The REE patterns have been used (together with other elements) for wine geographical origin authentication (Greenough et al., 1997; Cattarino et al., 2011). Furthermore, the addition of low levels of REEs may promote the productivity of plants and lead to accumulation in soils and plants with the consequent risk toxic risk to human body (Bertoldi et al., 2009). It is not weird then that due to the ever-increasing indirect use of REEs in agriculture (Liang et al., 2005), these elements are gaining attention due to rapid rise of modern industries and technological use (they are used in radar systems, rechargeable batteries, mobile phones, etc.; Hu et al., 2006; Ramos et al., 2016). These industrial uses can ultimately cause the accumulation of significant amounts of REEs in the soils (Otero et al., 2005). It is not uncommon for it drawing attention towards potential risks of intensive industrialization and modern agriculture to the exposure of REEs.

Viticulture is one of the mainstays of the farming economy in La Mancha (Central Spain). In this region, the PDO Valdepeñas (which occupies some 22,000 ha) produces 30% of the national wine production and it plays a significant social and economic role. The introduction of PDO Valdepeñas wines to markets in Asia and America, especially in the last decade, has reinforced the need to guarantee the quality and origin of the wine. Furthermore, since the territory affected by future mining is dedicated to vineyards, as is part of the study area, there is some social concern in this respect and it is necessary to explore the connection between vineyards and REE contents.

On the basis of the above, the main purpose of this study was to explore and obtain an overview of the levels and distribution patterns (under field study conditions) of some REEs (neodymium, cerium, lanthanum, yttrium, and scandium) in calcareous vineyard soils located in the PDO Valdepeñas. The possible enrichment in these elements was also evaluated. The final goal was to obtain relevant information on pedogeochemical quality (in the context of a semi-arid Mediterranean environment) for one of the most extensive and traditional wine regions in the world, La Mancha.

## Material and methods

### Study area

**Fig. 1:**
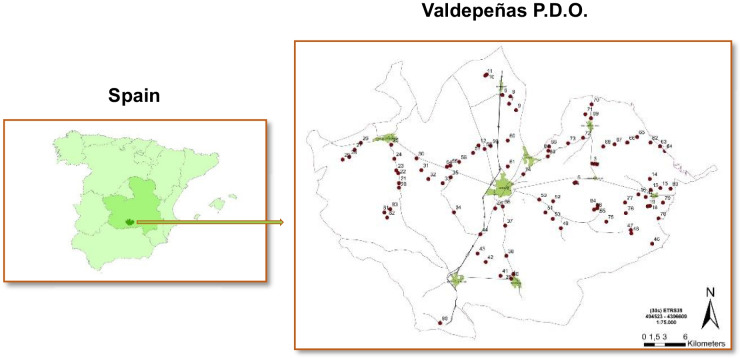
Geographical location of the study area (left) and sampling points in red (right)

The study area is located in the province of Ciudad Real, in central Spain, specifically within the PDO “Valdepeñas.” This territory extends over several municipalities (Alcubillas, Moral de Calatrava, San Carlos del Valle, Santa Cruz de Mudela, Torrenueva, and Valdepeñas) and partially over four other municipal areas (Alhambra, Granátula de Calatrava, Montiel, and Torre de Juan Abad, Fig. 1). The area occupies a flat territory based on tertiary and quaternary materials (made up of a wide range of carbonate materials, including marl, limestone, and various sediments), on which residual reliefs of quartzites, schites, and slates (Paleozoic materials) stand out. Occasional volcanic spaces are present and, to a lesser extent, granitic zones.

The climate of the area is type Csa according to the Köppen–Geiger classification, which means that it is a “temperate” climate. According to the Papadakis classification, the zone has a “temperate Mediterranean” climate and the humidity regime in this region is “dry Mediterranean.” Specifically, the average annual temperature in the area is 14.6 °C, with an average temperature in the warmest month of 25.5 °C and 5.6 °C for the coldest month. The frost period lasts for 4–5 months. Regarding rainfall, the area has average values of 450 mm per year with an average dry period of 4–5 months.

Vineyards, cereals, olive trees, and irrigated orchards are the main agricultural activities in the region. Sloping areas and lithic soils maintain natural vegetation, mostly holm oak forest with degraded areas.

### Soil identification and sampling

In this study, which is part of a larger project, 90 geo-referenced soil profiles were selected and opened by a machine in order to obtain a full description according to FAO Guidelines (FAO, 2006). The selection and sampling of the profiles were based, amongst other factors, on the geomorphological variability, the spatial distribution of the parent materials and soil types, and also according to the distribution of land cultivated with vines in the PDO. In each profile, the different horizons were identified and sampled for characterization and classification purposes. Given that the majority of the active vine roots are located at a depth aprox. of 10–80 cm, the samples were taken from the Ap and Bt or Bw or Ck horizons. The surface horizon (Ap 0–40 cm) is the top layer of the mineral soil horizons, in which most biological activity occurs; therefore, it contains more organic matter and less pedogenic material. In addition, it is the horizon where cultivation work is carried out and this management can be changed its original properties. The subsurface horizon (Bw 40–80 cm, Bt 40–80 cm, Ck > 80 cm, or Ckm > 80 cm) consists of mineral layers which are significantly altered by pedogenesis or by accumulation of other minerals (clay illuviation) or calcium carbonate that are migrating downwards from the topsoil horizons. Plant roots (such as vine) can penetrate throughout this layer. The sampling campaign for soil was carried out during 2018 and 2019. The dominant soil type and the profiles were selected based on the nature of the parent materials, slope, erosion, landform, land use, and the opinion of local farmers. The soil samples were taken from horizon to horizon separately from the parent material. Afterwards, the samples were sealed in plastic bags.

The macromorphological analysis of the 90 profiles described and sampled showed that the morphological features of soil profiles in PDO Valdepeñas are generally formed by a sequence of horizons of type Ap-Bt-Ckm, Ap-Bt-Ck, Ap-Bw-Bkm, Ap-Bw-Ck, Ap-Ckm type, or simply Ap-Bw-C or Ap-C-R. The soil orders identified are mainly Alfisols, Inceptisols, and Entisols according to Soil Taxonomy (Soil Survey Staff, 2014). These orders correspond to the soil groups Luvisols, Calcisols, Cambisols, Regosols, and Leptsols according to IUSS Working Group WRB. (2015). Calcisols represent the greatest proportion.

### Laboratory methods

After field sampling, all of the soil samples were dried at room temperature to a constant weight and sieved through a 2 mm mesh. The coarser material was discarded and the remaining fine-earth fraction was homogenized prior to analysis. Calcium carbonate was determined using a calcimeter.

X-ray fluorescence (XRF) spectrometers are widely used for the determination of elements with atomic numbers from 4 (beryllium) to 92 (uranium) at concentrations from 0.1 μg g^−1^ to high percentage levels (Fittschen & Falkenberg, 2011, El-Bahi et al., 2013; McComb et al., 2014). The content of analyzed REEs was determined using an X-ray fluorescence spectrometer (PHILIPS PW 2404) in solid mode on a powdered aliquot of each sample. The pearls of soil samples were analyzed with a maximum power of 4 kW (set of crystal analyzers for LiF220, LiF200, Ge, PET and PX1, flow detector, and twinkle detector). Quality control was achieved using Certified Reference Materials for soils (NIST 2710 and CRM 039). The analytical precision and accuracy were accepted only when RSD values were below 7% for the REEs (according to the result of duplicate measurements of all samples). The recoveries gave the overestimate compared to the certified values. The detection limits were (in mg kg^−1^) Nd 3.59; Ce 1.33; La 2.34; Y 0.31; and Sc 1.79.

There are different indexes generally used to identify metal concentrations of environmental concern like the geoaccumulation indexes (I-geo) and the concentration factor (C_f_). I-geo is a evaluating method of the degree of metal contamination in sediments proposed by Müller (1969). I-geo values were calculated as follows:

$${I}_{geo}={log}_{2}\frac{{C}_{n}}{1.5{B}_{n}}$$

where C_n_ represents the measured values and B_n_ is geochemical background of the site; the factor 1.5 was used to analyze natural fluctuations in the content of a given substance in the environment and to detect anthropogenic influences (Atiemo et al., 2011). The regional geochemical background values established by Jiménez-Ballesta et al. (2010) were used in the calculations. The seven categories of I-geo are shown in Table 1.

**Table 1:** Categories for the geocumulation index (I-geo), by Buccolieri et al. (2006), and the concentration factor (Cf)

Value	Classification
Geocumulation index (I-geo)	
I-geo ≤ 0	Uncontaminated
0 < I-geo < 1	Uncontaminated to moderately contaminated
1 < I-geo < 2	Moderately contaminated
2 < I-geo < 3	Moderadately to strongly contaminated
3 < I-geo < 4	Strongly contaminated
4 < I-geo < 5	Strongly to extremely contaminated
I-geo ≥ 5	Extremely contaminated
Concentration factor	
0 ≤ Cf < 1	Low contamination factor
1 ≤ Cf < 3	Moderate contamination factor
3 ≤ Cf < 6	High contamination factor
6 ≤ Cf	Very high contamination factor

A quantitative contamination index was also used, namely the concentration factor (C_f_), which is defined as follows:

$${C}_{f}^{i}=\frac{{C}_{0-1}^{i}}{{C}_{n}^{i}}$$

where C^i^_f_ is the ratio obtained by dividing the mean concentration of each metal in the soil (C^i^_0–1_) by the baseline or background value (concentration in unpolluted soil, C^i^_n_) (Abrahim & Parker, 2008). The four categories of concentration factor C_f_ are shown in Table 1.

### Statistical analysis

Statistical analysis of the data was carried out using Microsoft Office Excel 2013 and the software Statistical Package for Social Science (SPSS 19.0 for Windows, SPSS Inc., IL, USA), both under institutional licenses for the University of Castilla-La Mancha (Spain). Predictive soil maps suffer from different types of errors, where the most common error sources could be the measurements, digitization, typing, interpretation, classification, generalization, and interpolation. Most of these approaches apply a probabilistic framework within which the soil attribute of interest at a single location is regarded as a realization of a random variable. The most commonly applied geostatistical approach is the kriging and IDW (inverse distance weighting). IDW allows appropriate control of parameters and therefore provides results with greater reliability, so we used IDW.

## Results and discussion

### REE concentrations

The data in Table 2 provide the total contents of REEs and calcium carbonate. The average content of Ce in surface horizons was 70.6 mg kg^−1^, with a standard deviation of 33.7; the minimum value found was 18.8 mg kg^−1^ and the maximum value was 227.8 mg kg^−1^. The average Ce content at depth was 67.6 mg kg^−1^ with a standard deviation of 59.4; the values obtained in this subsurface horizon ranged from 7.0 to 432.1 mg kg^−1^. The standard deviation and kurtosis values are quite high (mainly in the subsurface horizon), and therefore, there is a marked variability in the contents of this element. Values in soils of 31 mg kg^−1^ have been cited by Kabata-Pendias (2010) and 38 mg kg^−1^ in Montiel (Central Spain) according to Castillo et al. (2003). Tyler (2004) reported Ce values of 66 mg kg^−1^ in rocks, 40 mg kg^−1^ in Japanese soils, 86 mg kg^−1^ in Chinese soils, and 11–68 mg kg^−1^ in Swedish topsoil. Salminen et al. (2005) obtained a value of 63 mg kg^−1^ for Spain (Table 3). Comparison of the values obtained and those of several countries in the same table leads to the conclusion that they are similar (or higher) in the surface horizons for the PDO Valdepeñas soils. The values obtained are higher than those found by Jimenez-Ballesta et al. (2010) for Castilla-La Mancha (the region in which the PDO Valdepeñas is located). These authors proposed a lower reference value (38 mg kg^−1^) so that some values for Ce can be considered to be close to pollution levels, but this point must be confirmed when more data is available.

**Table 2:** Mean concentrations of calcium carbonate and REEs, expressed on the basis of dry matter; mean, standard deviation (s.d.), minimum (Min) and maximum (Max) of REEs elements (in mg·kg^−1^). N number of samples

**Type of horizon**		**CaCO**_**3**_	**Ce**	**Nd**	**La**	**Y**	**Sc**
**Surface** **(Ap)**	***N***	90.0	90.0	90.0	90.0	90.0	90.0
	**Mean**	17.1	70.6	32.9	36.2	21.6	13.7
	**Stad Desv**	13.1	33.7	15.9	15.4	5.1	3.0
	**Min**	0.0	18.8	9.7	6.6	8.9	6.3
	**Max**	57.8	227.8	115.9	99.2	33.9	19.8
	**Kurtosis**	0.0	6.0	9.1	3.3	− 0.3	0.0
**Subsurface** **(B or C)**	***N***	81.0	81.0	81.0	81.0	81.0	81.0
	**Mean**	23.1	67.6	31.8	34.4	19.6	13.9
	**Stad Desv**	19.0	59.4	28.8	27.6	6.9	4.2
	**Min**	0.1	7.0	5.2	3.9	5.5	4.6
	**Max**	66.7	432.1	203.9	202.6	35.7	22.3
	**Kurtosis**	−0.5	22.1	23.9	20.5	−0.5	−0.4
**Enrichment factor** **(A/B or A/C)**		0.74	1.04	1.04	1.05	1.10	0.98

**Table 3:** REE concentrations (mg kg^−1^) in this study and various countries of the world according to different authors

		**Average concentrations (mg kg**^**−1**^**)**				
Country	**Authors**	**Ce**	**Nd**	**La**	**Y**	**Sc**
Germany	Hu et al., 2006 Salminen et al., 2005- El-Ramady, 2010-	5.92 48	2.53 20	- 23	-	- 6.1
Japan	Hu et al., 2006 Yoshida et al., 1998	39.80 40	17.60 18	- 18	-	-
Australia	Hu et al., 2006 Diatloff et al., 1996	60.49 60	14.63 15	15	5.3	-
China	Hu et al. 2006 Lijun et al., 2004-Li X et al., 2013- Zhang et al., 2008 Zhang et al., 2008 Wei et al., 1991	77.32 67 95.5 64.7	29.31 26 30.5 25.1	- 67 41.3 37.4	- 27	- 12
EU	Hu et al., 2006 Laul et al., 1979	25.67 67	9.98 30	- 30	- 16	- 9.9
Brasil	Oliveira et al., 2012 Silva et al., 2016	82 43.5	25 17.7	20 20.8	7.8 4.45	18 3.31
France	Salminen et al., 2005	63	27	31	9.3	9.3
Sweden	Sadeghi et al., 2013	37.8	15.1	17.4		
Spain	Salminen et al., 2005 Jiménez-Ballesta et al., 2010	63 57.7	28 21.6	33 23.6	-	10 23.6
Spain	In this study	70.6–67.6	32.9–31.8	31.8	21.6–19.6	14.0

The Y content ranged between 8.9 and 33 9 mg·kg^−1^, with an average value of 21.6 mg kg^−1^ for the surface horizon, while for the subsurface horizon, the Y content varied between 5.5 and 35.7 mg kg^−1^ with a mean value of 19.6 mg kg^−1^. Tyler (2004) reported Y values in the earth’ crust of 31 mg kg^−1^, in soils in China 22 mg kg^−1^ and in topsoil in Sweden of 4.9–17.6 mg kg^−1^. The Y contents of the reference soils are markedly different from the Y contents in Australia, France, and Brazil (Table 3), and therefore, the PDO Valdepeñas soils can be characterized by normal to moderately higher Y contents.

The average content of Nd in the surface horizons was 32.9 mg kg^−1^, with a standard deviation of 15.9; the minimum value found was 9.7 mg kg^−1^ and the maximum value was 115.9 mg kg^−1^. The average content of Nd at subsurface horizon was 31.8 mg kg^−1^ with a standard deviation of 28.8; the values obtained in this subsurface horizon ranged from 5.2 to 203.9 mg kg^−1^. The values found in several other countries (Table 3) did not exceed 30 mg kg^−1^.

Regarding the lanthanum content, the mean value was 36.2 mg kg^−1^; with a typical deviation of 15.4 mg kg^−1^, the minimum was 6.6 mg kg^−1^ and the maximum 99.2 mg kg^−1^ (this latter value could be the result of anthropic contamination). In addition, the value of 202.6 mg kg^−1^ in a subsurface horizon suggests that these samples were obtained from polluted locations. The mean values and standard deviations in the two horizons are similar. Tyler (2004) reported La values of 35 mg kg^−1^ in rocks, 18 mg kg^−1^ in Japanese soils, 44 mg kg^−1^ in Chinese soils, and between 5.5 and 33.2 mg kg^−1^ in Swedish soils. Therefore, in general terms, the soils in the study area have normal La values, apart from the exceptionally high values mentioned above.

Finally, the Sc concentration mean values are 13.7 mg kg^−1^ and 13.9 mg kg^−1^ in surface and subsurface horizons, respectively, with a maximum value of 19.8 mg kg^−1^ in the Ap horizon and a minimum of 4.6 mg kg^−1^ in the subsurface horizon. These results are within the concentration range of Sc found in soils (Hu et al., 2004; Tyler, 2004).

In general, standard deviations of the elements up to 33.7 for surface horizons and 59.4 for subsurface horizons were obtained. The kurtosis data sets (a measure of whether the data peak sharply or flatten out relative to a normal distribution) show that three elements (Nd, Ce, and La) have high kurtosis (i.e., they tend to a distinct peak close to the mean, decline rather rapidly, and have heavy tails), while another two elements (Y and Sc) have low kurtosis (i.e., they tend to flatten close to the mean rather than have a sharp peak). Indeed, the values obtained for the kurtosis of Y and Sc are very low, and are even lower than the critical values; for this reason, these distributions can be fitted to a normal learning curve.

Analysis of all these data leads to the conclusion that the concentrations vary by several orders of magnitude, with their mean values within the normal ranges reported worldwide (Table 3, Laul et al., 1979; Wedepohl, 1995; Diatloff et al., 1996; Lijun et al., 2004; Salminen et al., 2005; Li et al., 2008; Zhang et al., 2008; El-Ramady, 2010; Kabata Pendias, 2010; Oliveira et al., 2012; Cheng et al., 2012; Li et al., 2013; Dolegowska et al., 2013). The values are also similar to, or slightly higher than, the reported regional data in this region (Jiménez-Balleta et al., 2010; Bravo et al., 2019; Higueras et al., 2020).

Diaz et al. (2013) found that in Ck horizons, the highest contents of REEs corresponded to the stable interfluvial planes of a lower basin (La 25.2 mg kg^−1^, Ce 41 mg kg^−1^, Nd 28 mg kg^−1^). According to the Chinese Environmental Monitoring Station (1990), the background values in soils are La 41.2, Ce 95.5, and Nd 30.5 (in mg kg^−1^). Pereira et al. (2019) reported the following values (mg kg^−1^) for a soil developed in Brazil for the A and C horizons, respectively: La (4.3/12.6), Ce (9.3/15.9), Nd (3.3/12.2), while in another profile, they quoted the following values: La (121.3/67.3), Ce (223.5/154.5), Nd (96.0/60.9). These horizons are therefore clearly different.

In fact, the detection of very high quantities of REEs at certain sampling points in the study area leads to the conclusion that anthropic mechanisms are in action. Thus, values of 432.1 mg kg^−1^ for Ce, 203.9 mg kg^−1^ for Nd, 202.6 mg kg^−1^ for La, 35.7 mg kg^−1^ for Y, and 22.3 mg kg^−1^ for Sc were determined. These anomalies can be explained, for instance, by mining activities, the possible release of industrial waste containing different heavy metal REEs or energy production industries (Xie et al., 2014). The possibility of relating the increase in concentrations of REEs in a few horizons with such industrial activities is remote, simply because in the territory that covers the PDO Valdepeñas, the industrial activity is essentially limited to wine-making. Therefore, the traditional application of phosphate fertilizers, which may contain REEs, is of particular importance (Kotelnikovaa et al., 2021; Lijun et al., 2004; Otero et al., 2005; Tyler, 2004; Volokh et al., 1990; Waheed et al., 2011). Specifically, Hu et al. (2004) indicated that phosphogypsum and phosphate fertilizers contain 27–45 mg kg^−1^ of La and 39–61 mg kg^−1^ of Ce.

**Fig. 2:**
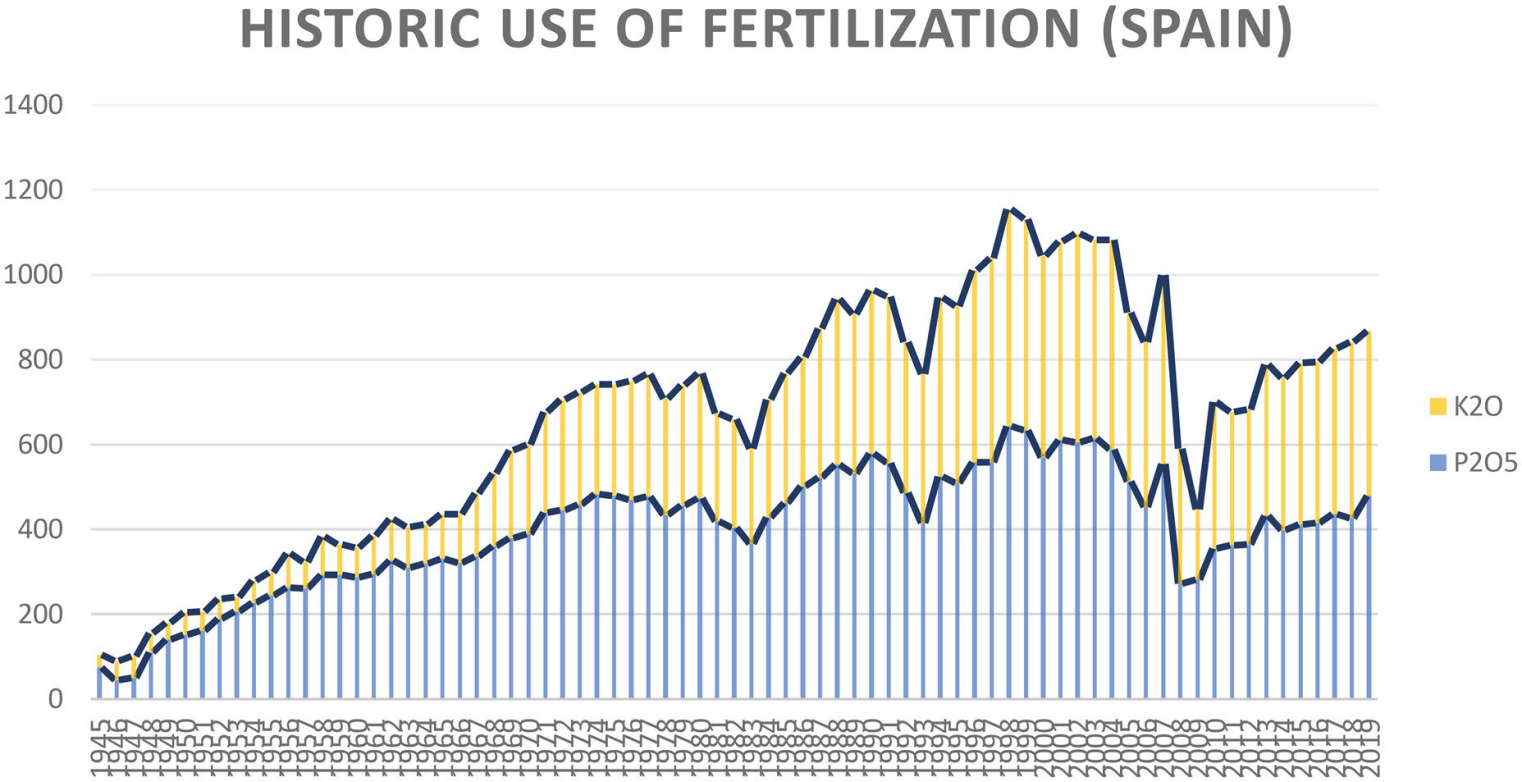
Graph showing the historical evolution of fertilizer use in Spain between 1945 and 2019, according to the Fertilizer Manufacturers Association

**Fig. 3:**
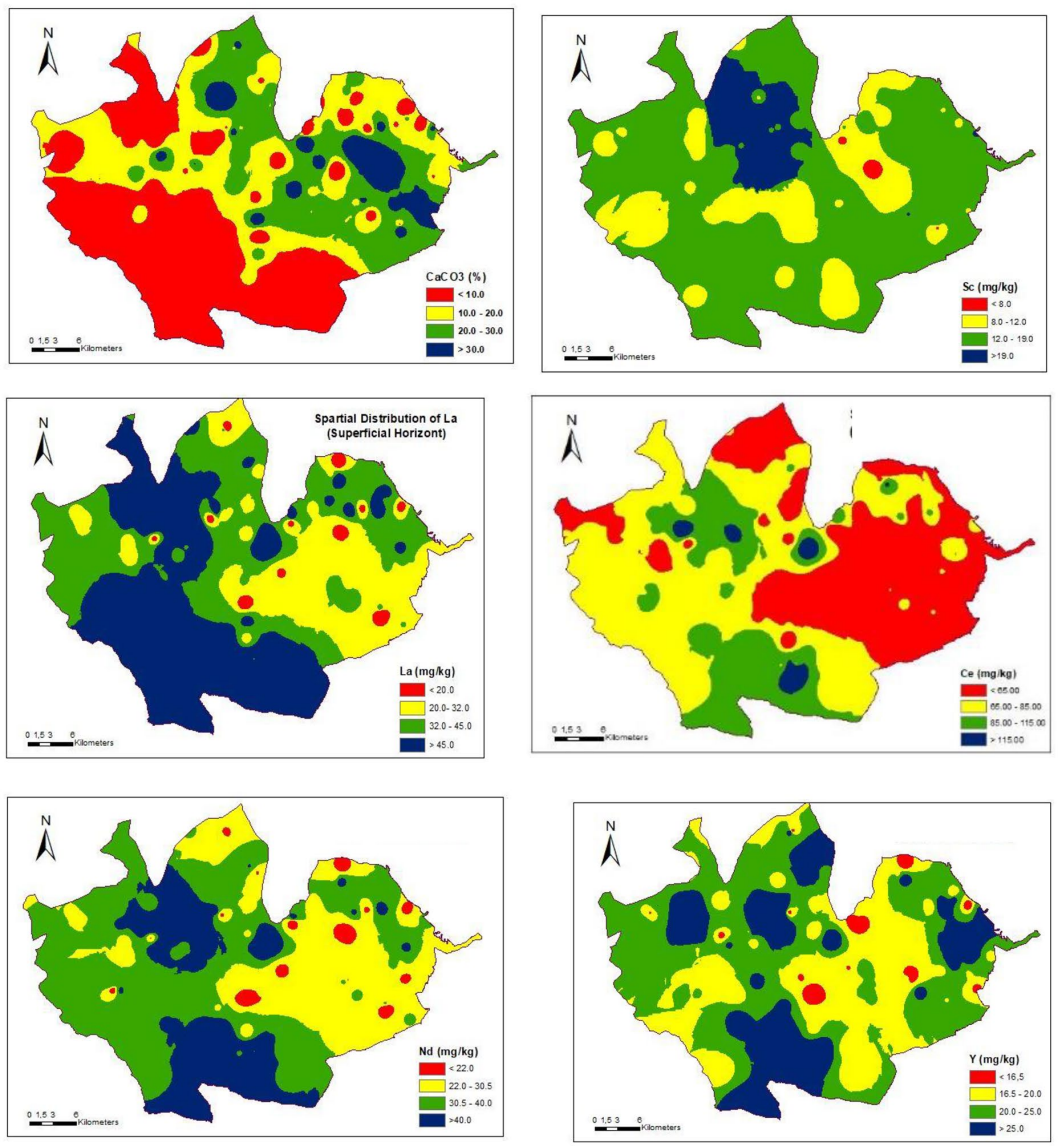
Spatial distributions of REEs in surface horizons

**Fig. 4:**
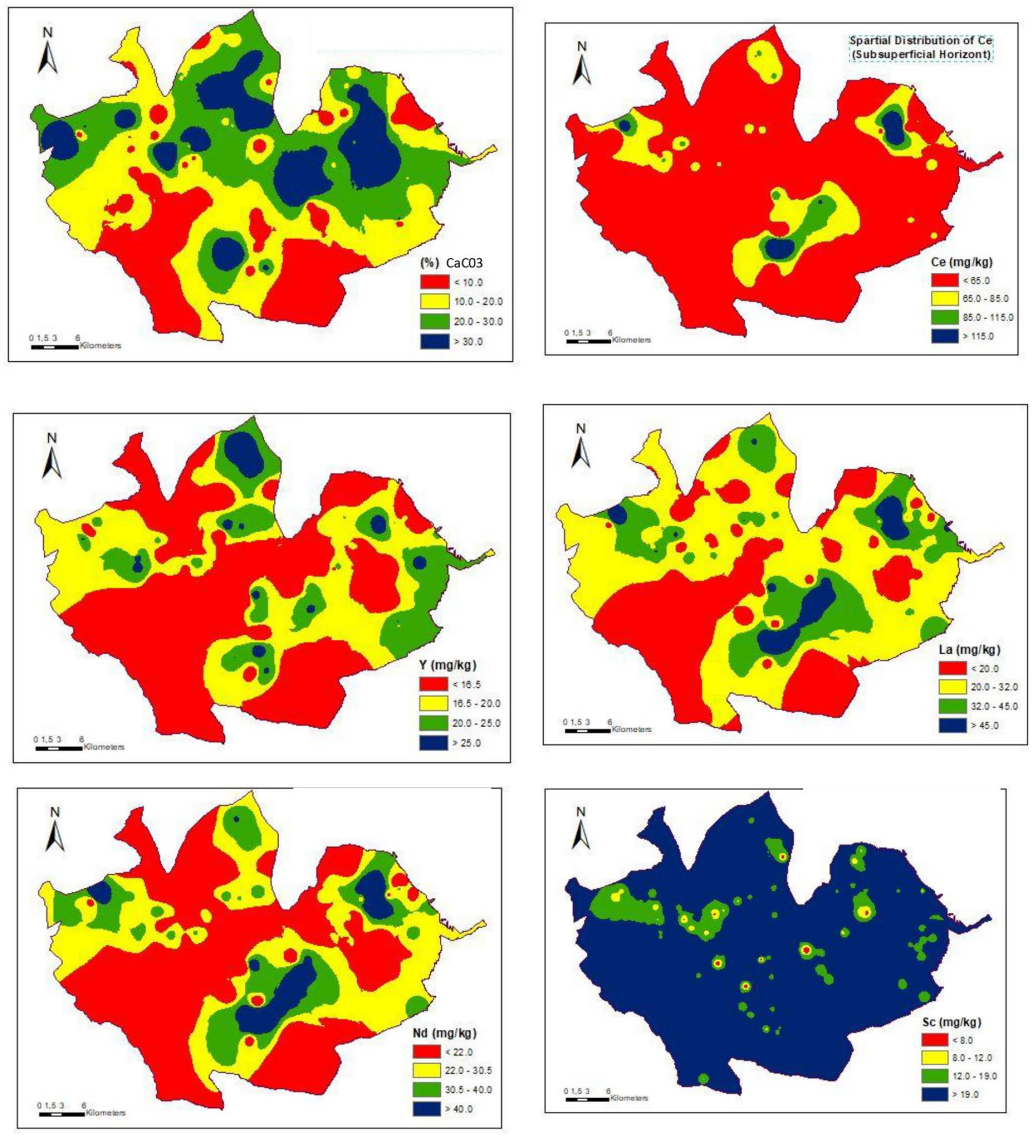
Spatial distributions of REEs in subsurface horizons

Indeed, the soils of PDO Valdepeñas, like those in the rest of Spain, have historically been fertilized with phosphates (Fig. 2) and the accumulation of REEs is to be expected, especially in the surface horizons. The fact that the accumulation is shown in a dispersive way (Figs. 3 and 4) can be explained by the random action commonly followed in fertilization.

### Assessment of pollution degree

The concentration factor (C_f_) and geo-accumulation index (I-geo) were used in this study to assess the potential contamination level of REEs in the soils; the results are summarized in Table 4. In general terms, although the soils studied are characterized by the absence of contamination by most of the elements analyzed, there is a gradient ranging from uncontaminated to moderate contamination (Håkanson, 1980; Müller, 1979; Yilgor et al., 2012). The I-geo of the mean values of all elements are uncotaminated, while La, Ce, and Nd appear as moderately contaminated related to máximum values. Regarding the contamination factor, Sc showed low contamination while Y, La, Ce, and Nd showed moderate contamination factor; only the máximum values of La, Ce, and Nd showed high contamination factor. These results indicate that only La, Ce, and Nd were significantly enriched in some surface soil samples (therefore only punctually).

**Table 4:** Concentration factors for elements (C_f_ concentration factor) and I-geo geoaccumulation index (max, maximum)

Element	Cf		I-geo	
	**Mean**	**Max**	**Mean**	**Max**
**Sc**	0.59	0.86	− 1.33	0.80
**Y**	1.13	1.78	− 0.39	0.25
**La**	1,48	3.96	− 0.05	1.40
**Ce**	1.17	3.79	− 0.35	1.39
**Nd**	1.43	5.03	− 0.06	1.74

### Spatial variability

In an effort to evaluate the relative enrichment or loss of the elements, the top/bot relationship was calculated. The relationships obtained (Table 2) show that the vertical distribution is controlled by the composition of the material and pedogenesis. Additionally, the surface enrichment at some points is indicative of contamination processes, although these soils cannot be classified as polluted.

The study of the spatial distribution of the contents in REEs is useful to evaluate and to search for areas with high REE contents. Several authors, such as Goovaerts and Webster (1994), and Rodríguez-Martín et al. (2006), have used the geostatistical approach for soil variation mapping. On applying this type of technique to the case in question here, specifically in relation to carbonates, the distribution maps for both surface and subsurface horizons have some similarities (Figs. 3 and 4) but, as one would expect, they are not exactly the same.

In the case of the spatial distribution map for cerium at the surface, there are four points that are consistent with possible sources of anthropic contamination and these points therefore carry a certain environmental risk. Three other points can be observed on the subsurface horizon but their coordinates do not coincide with those of the aforementioned surface sites. In the surface horizons, there is a large sector located to the southwest with the highest Ce values.

In the case of La, an irregular dotted area is observed both on the surface and subsurface horizons. In the surface horizon, there is a zone (in the SW direction) with the highest values and a relatively uniform appearance, but this does not coincide with the values for the subsurface horizon. In fact, in the subsurface horizon, there are hardly any high values at all. As a result, it can be assumed that there are some external contributions, but not with a consistent distribution. In the case of Y, there is a clear contrast between the spatial distributions in the surface horizon and the subsurface horizon, although in the distributions of both, there are dotted areas with dissimilar locations. The surface horizon has higher values and this could again be related to external contributions (probably the addition of fertilizers), as stated Nziguheba and Smolders (2008).

The case of Sc is very remarkable, since its values are higher in the subsurface horizon in almost all of the PDO, although these areas are dotted by numerous isolated points that have values (close to the maximum). In the surface horizon, the dominant values do not reach the maxima but rather the next rank in the established order. As a consequence, the influence of an external factor can be ruled out in this case. It can be concluded that, since the contents are lower at the surface than at depth, the origin of Sc is fundamentally lithoedaphological. Finally, in the surface horizons, Nd shows several scattered points and intermediate values predominate.

## Conclusions

The patterns of REEs and the accumulation of other similar elements in soils from vineyards of the PDO Valdepeñas have been evaluated. The results show mean values (mg kg^−1^) in surface horizons (Ap) of Ce 70.6, Nd 32.9, La 36.2, Y 21.6, and Sc 13.7, whereas in subsurface horizons (Bt or Bw and some Ck), the values are Ce 67.6, Nd 31.8, La 34.4, Y 19.6, and Sc 13.9. Standard deviations of up to 33.7 in surface horizons and 59.4 in subsurface horizons were determined. The elements can be separated into two groups on the basis of the kurtosis values: the first group consists of Nd, Ce, and La (with high kurtosis values) and the second group contains Y and Sc (with low kurtosis values). Based on the data obtained, the concentrations of REEs in our study were similar to those found in other vineyard soils.

REEs can be present in the soil as a product of the weathering of the natural rocks, or because they come as part of pollution loads generated by human activities. Soil assessments with concentration factor and geo-accumulation index indicate that the soils in PDO Valdepeñas are not contaminated with REEs, except in small enclaves. The spatial patterns of REEs were established methodically through geostatistical analysis and this showed spatial variations dotted with local variations, some of which are anomalies of anthropic origin. Given the spatial variability and its relationship with lithological materials and soil types, the contents of most of the individual REEs depend on the natural occurrence of parent material and soil-type circumstances. Only in some cases, the elevated detected concentrations of REEs suggest anthropogenic inputs, probably associated with agricultural techniques such as fertilization.

As a final conclusion, the results reported here provide baseline data for future geoaccumulation analysis of soils in the study area and similar agroecological vineyard zones. In addition, it can be deduced that the use of agrochemicals in this area is carried out in a manner that does not pose an ecological risk, with management practices that do not have a negative influence. However, it seems unlikely that REE pollution may cause some environmental problems in the near future, so it is suggested that further studies be undertaken to identify possible pollution at the local level within the area.

## Data Availability

Data will be made available on reasonable request.
